# Supplementary material for: Hourly data for evaluating the carbon dioxide emission factor of heat pumps or other devices connected to the Italian grid
Source: Data Brief. 2022 Oct 20;45:108682. doi: 10.1016/j.dib.2022.108682 (PMC9679705; doi:10.1016/j.dib.2022.108682)
Supplement: Supplementary file 1 [file mmc1.zip › DatainBrief.docx]

**Article information**

**Article title**

Hourly data for evaluating the carbon dioxide emission factor of heat pumps or other devices connected to the Italian grid

**Authors**

Paolo Valdiserri, Vincenzo Ballerini, Eugenia Rossi di Schio*.

**Affiliations**

Alma Mater Studiorum – University of Bologna. Department of Industrial Engineering DIN. Viale Risorgimento 2, I-40136 Bologna. Italy.

**Corresponding author’s email address and Twitter handle**

[eugenia.rossidischio@unibo.it](mailto:eugenia.rossidischio@unibo.it)

**Keywords**

Sustainability transitions; Carbon emission intensity; CO2 emissions; Energy transition; Decarbonization; Dynamic simulations

**Abstract**

This data article includes an elaboration of carbon dioxide data available from three different online sources in the years from 2016 to 2019. The data article refers to the paper “Interpolating functions for CO2 emission factors in dynamic simulations: the special case of a heat pump” by the same authors. The data are provided on an hourly basis and are useful to determine the carbon dioxide emission of an electric heat pump or other devices connected to the Italian grid. The importance of the provided data is related to the possibility of having an accurate estimation of the CO2 emission when the device works for only a limited period of time during the year or day. Moreover, since the given data are provided in electronic format (.txt file or .xlsx spreadsheet) they are very useful to perform dynamic simulation using self-made or commercial software such as Trnsys, Energy Plus etc.

**Specifications table**

| **Subject** | Renewable Energy, Sustainability and the Environment |
| --- | --- |
| **Specific subject area** | Data refers to the subject area of energy sustainability, in particular to carbon dioxide emissions estimation. |
| **Type of data** | Table |
| **How the data were acquired** | Primary data have been retrieved from three different sources: data on electric energy production and exchange with other Countries have been downloaded from Italian Transmission System Operator TERNA online platform, data on non-renewable carbon emission factor have been obtained from AIB (Association of Issuing Bodies) and from ISPRA (Italian Institute for Environmental Protection and Research). Some other data on carbon dioxide intensities have been obtained by IPCC (Intergovernmental Panel on Climate Change) yearly reports.  All data employed are freely available online.  Collected data have been analyzed and elaborated by means of spreadsheets. |
| **Data format** | Analyzed |
| **Description of data collection** | Primary data on hourly electricity production and exchanges with other countries have been downloaded from TERNA platform by means of the online platform available on TERNA website.  Data on annually non-renewable emission factors have been obtained from ISPRA annual report on atmospheric emission factors of greenhouse gas; other data on emission factors have been obtained from annual report of AIB and also from IPCC reports. |
| **Data source location** | Data on electricity production and exchanges with other Countries:   - Institution: TERNA S.p.A (Italian Transmission System Operator) - City: Rome - Country: Italy - Data available online: <https://www.terna.it/it/sistema-elettrico/transparency-report/download-center>   Data on annual grid losses in Italian power grid:   - Institution: TERNA S.p.A (Italian Transmission System Operator) - City: Rome - Country: Italy - Data available online: https://www.terna.it/it/ istemaelettrico/statistiche/pubblicazioni-statistiche   Data on annual non-renewable carbon dioxide emission factor for Italy:   - Institution: ISPRA (Italian Institute for Environmental Protection and Research) - City: Rome - Country: Italy - Data available online: <https://www.isprambiente.gov.it/en/databases/data-base-collection/environmental-indicators>   Data on carbon dioxide emission factors by renewable source:   - Institution: IPCC (Intergovernmental panel on Climate Change) - Data available online: https://www.ipcc.ch/report/ar5/syr/   Data on annual non-renewable carbon dioxide emission factor for European Countries:   - Institution: AIB (Association of Issuing Bodies) - City: Bruyelle - Country: Belgium - Data available online: <https://www.aib-net.org/facts/european-residual-mix> |
| **Data accessibility** | Dataset available online: Ballerini, Vincenzo; Rossi di Schio, Eugenia; Valdiserri, Paolo (2022), “Hourly carbon dioxide emission factors for Italy”, Mendeley Data, v1  http://dx.doi.org/10.17632/5dt2mf8mnt.1 |
| **Related research article** | P. Valdiserri, V. Ballerini, E. Rossi di Schio, Interpolating functions for CO2 emission factors in dynamic simulations: the special case of a heat pump, Sustainable Energy Technologies and Assessments, (under review) |

**Value of the data**

- Data on hourly emission factors are useful because allows to determine accurately the emission of an electric device connected to power grid, for example a heat pump
- The data can be used by researchers that investigate the carbon dioxide emissions due to the electric devices connected to the power grid
- The data can be imported in any spreadsheet for hourly evaluation of carbon dioxide emissions. Data can also be used directly in commercial dynamic simulation software packages such as Trnsys, Energy Plus, etc.
- Possible future development is the determination of the emission factors for other Countries (in fact the dataset refers on to Italian scenario)

**Data description**

The dataset is provided in format (.xlsx) and (.txt).

The first column refers to the hour of the year (1 – 8760 h); the other five columns refer to the value of the hourly emission factor expressed in kg/kWh. The data have been determined according to the model expressed in the next section. The emission factors have been determined for Italy over a period of 4 years, from 2016, to 2019. The last column in the sheet refers to an hourly averaged emission factor, that takes into account the four-year period 2016-2019.

**Experimental design, materials and methods**

The hourly emission factor *EF_h_* (kg/kWh) that refers to the the h-th hour of the year, have been determined according to the following equation, which also describes the model adopted

| ${EF}_{h}=$ $\frac{EE_{nren,h}\cdot EF_{nren}+\sum_{j} EE_{ren,j,h}\cdot EF_{j}+\sum_{k} EE_{imp,k,h}\cdot EF_{k}-m_{int+imp,h}\cdot\frac{EE_{exp,h}}{EE_{int+imp,h}}}{\sum_{k} EE_{imp,k,h}+\sum_{j} EE_{ren,j,h}+EE_{nren,h}-EE_{exp,h}}p$ | (1) |
| --- | --- |

Where:
*EE_nren,h_* (kWh) is the hourly electricity produced in Italy from non-renewable sources;
*EF_nren_* (kg/kWh) is the yearly carbon dioxide emission factor from non-renewable (source ISPRA);
*EE_ren,j,h_* (kWh) is the hourly electricity produced in Italy by the j-th renewable source (geothermal, wind, photovoltaic, bio-energies and hydropower), source TERNA;
*EF_j_* (kg/kWh) is the emission factor related to the j-th renewable source (source IPCC);
*EE_imp,k,h_* (kWh) is the hourly electricity imported from the k-th Country (Italian power grid is connected to 7 Countries: France, Switzerland, Austria, Slovenia, Montenegro, Malta and Greece), source TERNA;
*EF_k_* (kg/kWh) is the annual emission factor related to the k-th Country (source AIB);
*EE_exp,h_* (kWh) is the hourly electricity exported from Italy to other Countries (source TERNA);
*m_int+imp,h_* (kg) is the mass of carbon dioxide related to electricity produced and imported in Italy;
*EE_int+imp,h_* (kWh) is the hourly sum of electricity produced and imported in Italy (source TERNA);
*p* (-) is the coefficient for grid losses (source TERNA).

**Ethics statements**

Only primary data from datasets publicly available have been used for the development of the present dataset. All the sources have been mentioned.

**CRediT author statement**

Paolo Valdiserri, Vincenzo Ballerini and Eugenia Rossi di Schio: conceptualization, methodology, writing – original draft;
Vincenzo Ballerini: data curation;
Paolo Valdiserri, and Eugenia Rossi di Schio: writing – review and editing.

**Acknowledgments**

This research did not receive any specific grant from funding agencies in the public, commercial, or not-for-profit sectors.

**Declaration of interests**

The authors declare that they have no known competing financial interests or personal relationships that could have appeared to influence the work reported in this paper.

**References**

- P. Valdiserri, V. Ballerini, E. Rossi di Schio, Interpolating functions for CO2 emission factors in dynamic simulations: the special case of a heat pump, Sustainable Energy Technologies and Assessments. 53 (2022) 102725. <https://doi.org/10.1016/j.seta.2022.102725>.
